# Draft genome sequence of a multidrug-resistant *Klebsiella pneumoniae* fecal isolate from a cow with clinical mastitis

**DOI:** 10.1128/MRA.00730-23

**Published:** 2023-10-30

**Authors:** M. Nazmul Hoque, Golam Mahbub Faisal, Zannatara Moyna, Md. Sayedul Islam, Ziban Chandra Das, Tofazzal Islam

**Affiliations:** 1 Molecular Biology and Bioinformatics Laboratory, Department of Gynaecology, Obstetrics and Reproductive Health, Bangabandhu Sheikh Mujibur Rahman Agricultural University (BSMRAU), Gazipur, Bangladesh; 2 Department of Microbiology and Public Health, BSMRAU, Gazipur, Bangladesh; 3 Institute of Biotechnology and Genetic Engineering (IBGE), BSMRAU, Gazipur, Bangladesh; University of Maryland School of Medicine, Baltimore, Maryland, USA

**Keywords:** whole genome, bovine mastitis, pathogenic, multidrug-resistant, *Klebsiella pneumoniae*

## Abstract

*Klebsiella pneumoniae* is one of the most important mastitis-causing pathogens. The multidrug-resistant *K. pneumoniae* strain MNH_G2C5F was isolated from the feces of a cow with clinical mastitis. The MNH_G2C5F strain had a genome size of 5,381,832 bp (85.0× coverage) and typed as sequence type 273 (ST273).

## ANNOUNCEMENT

*Klebsiella pneumoniae* is one of the most important emerging opportunistic pathogens causing bovine clinical mastitis (CM) (1 – 3). Mastitis caused by *K. pneumoniae* is characterized by the overwhelming immune response with severe clinical episodes and a considerable decrease in milk production (4). Furthermore, control of this pathogen is impaired by multidrug-resistant phenotype and genotype, representing a global threat to dairy holders (5, 6). Due to its clinical importance and increasing antimicrobial resistance (7 – 9), *K. pneumoniae* is one of the main foci of surveillance efforts and molecular epidemiology studies in bovine mastitis.

For isolation and phenotypic identification of *K. pneumoniae*, fecal sample from a CM cow was collected, homogenized in distilled water (1:1 ratio), streaked onto MacConkey agar (Oxoid, Thermo Scientific, UK) plates, and incubated at 37°C for 24 h (2, 10). Gram-negative bacilli were recovered and identified as *K. pneumoniae* by biochemical tests (e.g., indole, methyl red, citrate, and lactose fermentation) (2, 11). The 16S rRNA gene was amplified using 27F (5′-AGAGTTTGATCCTGGCTCAG-3′) and U1492R (5′-CTACGGCTACCTTGTTACGA-3′) primers (12), and DNA sequencing was performed under Illumina MiSeq platform (Illumina Inc., San Diego, CA, USA) for further confirmation. The confirmed *K. pneumoniae* strain MNH_G2C5F (GenBank accession no. OR405045) was further grown in nutrient broth (Biolife Italiana, Italy) at 37°C for 24 h, and genomic DNA was extracted from the broth culture using the QIAamp DNA mini kit (Qiagen, Hilden, Germany). NanoDrop 2000 UV-Vis Spectrophotometer (Thermo Fisher, Waltham, MA, USA) was used to check the concentration and quality of DNA. DNA libraries were prepared using the Nextera DNA Flex library preparation kit (Illumina, San Diego, CA, USA) according to the manufacturer’s protocol, and whole genome sequencing (WGS) of the prepared libraries was performed with the Illumina MiSeq sequencer using a paired-end (2 × 250 bp) protocol. The paired-end raw reads (*n* = 12,799,136 bp) were trimmed using Trimmomatic v.0.39 (13) to remove Illumina adapters, known Illumina artifacts, and phiX and quality checked using FastQC v.0.11.7 (14). Reads with Phred score >20 were assembled *de novo* using SPAdes v.3.9.0 (15), and the quality of the assembled genome was checked using QUAST v.5.0.2 (16). The NCBI Prokaryotic Genome Annotation Pipeline v.6.4 (17) was used for genome annotation, and BacWGSTdb 2.0 (18) was used for sequence typing. ResFinder 4.0 (19), PlasmidFinder (20), VFDB (21), and RAST FIGfams v.70 (22) databases were used to predict antimicrobial resistance genes (ARGs), plasmid, virulence genes, and metabolic functions, respectively, in the draft genome. Default parameters were used for all software unless otherwise specified.

The MNH_G2C5F genome is estimated to be 5,381,832 bp, with 85.0× coverage and 65 contigs. The chromosome length, GC content, RNA genes, and *N*_50_ value of the assembled genome were 5,380,166 bp, 57.4%, 87, and 260,342 bp, respectively. The MNH_G2C5F strain was typed as *K. pneumoniae* sequence type 273 (ST273) according to seven-gene multilocus sequence typing (MLST) (*gap*A, *inf*B, *mdh*, *pgi*, *pho*E, *rpo*B*,* and *ton*B) on cgMLST scheme (18). The draft genome contained 395 subsystems and 5,311 protein-coding sequences. Fifty-one ARGs and 60 virulence genes were predicted in the draft genome. Four plasmid replicons such as IncFIA(HI1), IncFIB(K)(pCAV1099-114), IncFII(K), and IncHI1B(pNDM-MAR) were identified in the genome (with 95% identity and 60% coverage).

## Data Availability

The 16S rRNA and draft genome sequences of *Klebsiella pneumoniae* strain MNH_G2C5F have been deposited at GenBank under the accession numbers OR405045 and JAULSG000000000. The raw Illumina reads have been deposited in NCBI SRA database under accession number SRR25276878 and BioProject accession number PRJNA994715.
